# The incidence of malignancies in asbestosis with chrysotile exposure: a large Chinese prospective cohort study

**DOI:** 10.3389/fonc.2023.1172496

**Published:** 2023-07-06

**Authors:** Jingwei Wang, Xiaoyun Huang, Ruimin Ma, Qian Zhang, Na Wu, Xuqin Du, Qiao Ye

**Affiliations:** ^1^ Department of Occupational Medicine and Toxicology, Clinical Center for Interstitial Lung Diseases, Beijing Institute of Respiratory Medicine, Beijing Chaoyang Hospital, Capital Medical University, Beijing, China; ^2^ Department of Respiratory Medicine, Civil Aviation General Hospital, Beijing, China

**Keywords:** asbestosis, lung cancer, standardized incidence ratio (SIR), malignancy, chrysotile

## Abstract

**Background:**

Asbestos exposure is closely related to the occurrence and development of various malignancies. This prospective cohort study aimed to evaluate the incidence rate and potential risk factors in a cohort of asbestosis patients in China.

**Methods:**

The incidence of malignancies was determined in patients who had been exposed to chrysotile asbestos and diagnosed with asbestosis sequentially at Beijing Chaoyang Hospital from 1 January 2007 to 31 December 2019. Cox regression analyses were used to analyze the correlations between clinical variables and asbestosis combined with malignancies.

**Results:**

A total of 618 patients with asbestosis were identified, of whom 544 were eligible for analysis. Among them, 89 (16.36%) were diagnosed with various malignancies. The standardized incidence ratios (SIRs) of patients with asbestosis combined with malignancies were 16.61, 175, 5.23, and 8.77 for lung cancer, mesothelioma, breast cancer, and endometrial carcinoma, respectively. The risks of all malignancies and lung cancer increased with initial exposure before 17 years old, longer asbestos exposure, and smoking.

**Conclusions:**

The SIRs of patients with asbestosis-related malignancies were significantly increased in lung cancer, mesothelioma, breast cancer, and endometrial carcinoma in a hospital-based Chinese cohort. Smoking and the duration of asbestos exposure increased the risk of lung cancer.

## 1 Introduction

Despite the well-documented health risks associated with asbestos, it is still widely used in various industries such as construction, insulation, and textiles due to its advantageous characteristics of durability and resistance to heat, chemical, and biological degradation (1). The global burden of disease related to occupational and environmental exposure to asbestos may have been significantly underestimated (1). The recognition of asbestos-related diseases (ARDs) is warranted especially in the countries and regions where the exploitation and application of asbestos have not been prohibited. By the end of 2013, more than 50 countries and regions, including all member states of the European Union, have banned the use of all forms of asbestos, including chrysotile (2). The World Health Organization estimated that 107,000 global annual deaths were due to ARDs, with 12,000 to 15,000 deaths in the USA (3). China is the third-largest asbestos reserve country worldwide and also the world’s top chrysotile consumer and second-largest producer. Over a million people may be occupationally exposed to asbestos in China, yet the disease statistics were underestimated, and the national burden of ARDs was not well known (4).

Since the beginning of the 20th century, inhalation of asbestos, including chrysotile, has been considered responsible for a number of non-malignant diseases, such as small airway dysfunction, chronic obstructive pulmonary disease, pleural fibrosis (plaque and thickening), and asbestosis (5–8). Asbestosis is a diffuse interstitial pulmonary fibrosis with impaired gas exchange and reduced lung capacity, causing progressive dyspnea and even respiratory failure. Malignancies can occur after exposure to asbestos in the absence of asbestosis. Exposure to chrysotile has been verified to increase the carcinogenicity risk of peritoneal and pleural malignant mesothelioma, lung cancer, and ovarian cancer (9, 10). More attention is currently being focused on other malignancies, including breast and endometrial carcinoma (11, 12).

The carcinogenic potency of short chrysotile and amphibole asbestos (crocidolite and amosite) fibers has been debated over the years. The data from the large database of epidemiology, toxicology, and *in vitro* research have shown that chrysotile increased the malignancies as well (13, 14). The purpose of this study was to evaluate the standardized incidence ratio of malignancies in asbestosis patients exposed to chrysotile and to identify the risk factors for cancer in a Chinese cohort of patients with asbestosis.

## 2 Methods

### 2.1 Study design

This descriptive study adopted a prospective cohort design and followed guidelines established by the Strengthening the Reporting of Observational Studies in Epidemiology (STROBE) checklist.

### 2.2 Settings and participants

The incidence of malignancies was determined in a hospital-based cohort of patients with asbestosis. Beijing Chaoyang Hospital had an occupational medical center, which is located 20 km away from the asbestos products factories opened from the 1950s to 1970s. Further details about the hospital selection are presented in the **Supplementary Material**. Patients, aged 18 years or older, who had been exposed to chrysotile asbestos and diagnosed with asbestosis at Beijing Chaoyang Hospital at any time from 1 January 2007 to 31 December 2019 were recruited. Patients with missing clinical data or other subtypes of pneumoconiosis, family history of lung fibrosis, or other interstitial lung diseases were excluded. The follow-up of each patient started at the beginning of the study period or first diagnosis and ended at the time of finding malignancies after exposure to chrysotile, loss to follow-up, or end of the study. The patients visited the clinic every 12 months, and the end of follow-up was set as 31 December 2021.

The diagnosis of lung cancer and mesothelioma related to asbestos was based on the Helsinki criteria for occupational malignancies (15). The diagnosis of other cancers is consistent with the latest guidelines, which are shown in the **Supplementary Material**.

### 2.3 Study procedure

#### 2.3.1 Data collection

Data were collected from the predesigned charts, which included demographics, anthropometric measurements, employment history, smoking status, alcohol consumption, medicine use, personal medical history, malignancy diagnosis status, and asbestosis diagnosis status.

Employment history, including any previous career, was collected by questionnaire for all patients. Due to the lack of atmospheric measurements and detailed information on the frequency of asbestos exposure for each job, individual exposure was determined by the duration of employment (number of years). Latency was defined as the time interval from the beginning of occupational chrysotile exposure until the asbestosis diagnosis. Tobacco consumption was calculated as cumulative pack-years smoked. The type, location, and timing of the malignancy’s diagnosis were ascertained by reviewing pre-existing medical records or through interviews with the patient. The diagnostic criteria for all malignancies are presented in the **Supplementary Material**. All patients underwent chest radiography, high-resolution computed tomography (HRCT), and tests for pulmonary function and serum tumor markers. All medical records and chest images were reviewed by a multidisciplinary team to confirm the presence of features indicating asbestosis.

#### 2.3.2 Lung function test

The patients with asbestosis underwent pulmonary function tests according to the recommendations of the American Thoracic Society and European Respiratory Society (16). Trained technicians performed pulmonary function examination using spirometry, whole-body plethysmography, and single-breath diffusing capacity for carbon monoxide (DLCO SB). Respiratory physiologists were blinded to the participants’ group. Only technically acceptable and repeatable data were included in the data analyses. To avoid the influence of age, height, and weight, spirometry, total lung capacity (TLC), and DLCO SB were presented as percentages of the predicted.

#### 2.3.3 Imaging

Chest radiographs were obtained from each patient. Two occupational medicine experts independently evaluated all images and were blinded to the clinical information. All disagreements were resolved through consensus. The interobserver correlation was good, with a value of 0.82. Asbestosis was classified into three stages according to the density and distribution of small and large opacities on the posterior chest radiograph, using a national criterion on the diagnosis of occupational pneumoconiosis (17), which is in line with International Labour Organization (ILO) classification guidelines (18). All enrolled patients underwent chest HRCT with a 1-s scan time, 0.625-mm slice thickness, and 10-mm interval from the lung apex to the base, including both lungs in the field of view. Further details about the stages of the radiograph, including how the chest radiographs were performed, are presented in the **Supplementary Material**.

#### 2.3.4 Statistical analyses

SPSS Statistics v. 23 (IBM Inc., Chicago, IL, USA) and GraphPad Prism v. 8 (GraphPad Software, La Jolla, CA, USA) were used to perform statistical analyses and create plots. Patients with asbestosis were classified into two groups, with or without cancer, to analyze job exposure data, lung function values, and serum tumor markers. Patients with asbestosis were classified into three groups according to the stages of radiographs showing the severity of the disease to analyze alongside demographics, as shown in the **Supplementary Material**. The characteristics of patient groups were compared across groups using the chi-squared test when the expected count for each data was ≥5. Otherwise, Fisher’s exact test was used. The results were expressed as counts and percentages (for categorical variables). Continuous data were analyzed using the two-tailed Student’s t-test (for two-group comparisons) or analysis of variance (for three-group comparisons) and were expressed as mean ± standard deviation and/or interquartile range. Standardized incidence ratios (SIRs) were calculated according to the national cancer incidence rates for China (19). The other SIRs whose malignancies were not reported in the article were calculated according to the latest epidemiologic data in the Chinese population (20–24). The SIR was the observed number of cancers in the study population divided by the expected number of cases. The expected number of cases was calculated as the product of person-years of observation and cancer incidence rates from the reference population. The 95% confidence intervals (CIs) for the SIR were calculated based on the Poisson distribution. The influencing factors including smoking, body mass index (BMI), exposure time, and latency were tested using Cox regression in all malignancies or lung cancer to assess their independent relationships with asbestosis combined with malignancies and adjusted for age and sex. Multivariate-adjusted odds ratios (ORs) and CIs were calculated using Cox regression analysis. Statistical significance was set at p < 0.05.

## 3 Results

### 3.1 Demographics

**Figure 1:**
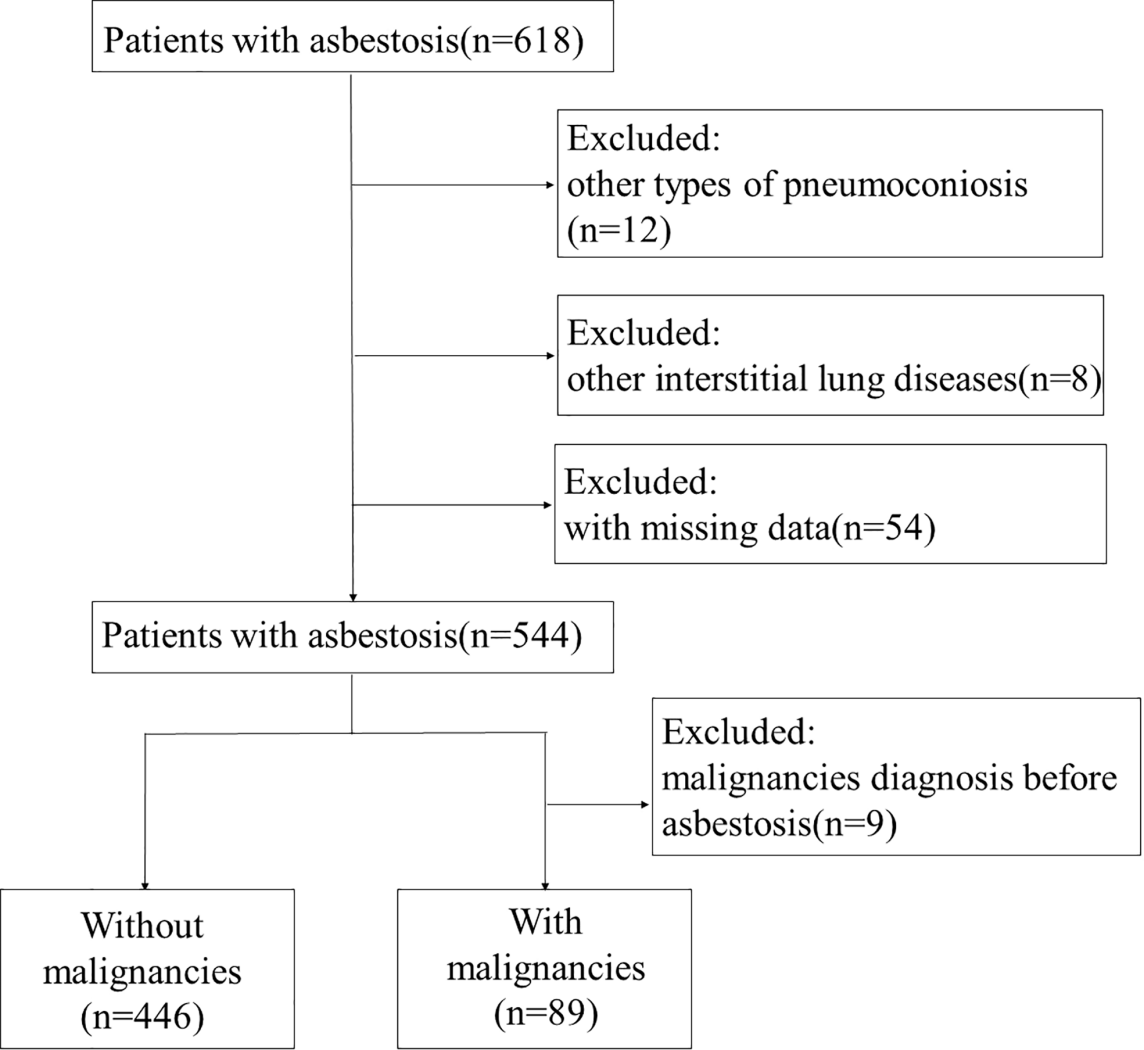
Flowchart of the study population. A total of 618 patients were recruited from Beijing Chaoyang Hospital. Of them, 12 patients were excluded due to diagnosis of other types of pneumoconiosis, 8 patients due to interstitial lung disease, and 54 patients due to missing data. Finally, 544 patients with asbestosis were included in the study. Nine patients were excluded from with malignancies group due to malignancy diagnosis before asbestosis.

In total, 618 patients with asbestosis were identified according to the diagnostic criteria for pneumoconiosis based on the ILO classification (18). Among them, 544 patients were eligible for analysis (**Figure 1**). For 544 patients, the mean age at entry to follow-up was 69.73 (SD 8.76) years, 240 (44.1%) were male, and 143 (26.3%) patients had a history of smoking (**Table 1**). In total, 89 (16.36%) patients had malignancies with a median of 10.0 (interquartile range (IQR) 6.0–14.0) years’ longitudinal follow-up. Of the 89 patients with malignant tumors of asbestosis, 27 patients (30.34%) were diagnosed with asbestosis and malignant tumors at the same time, and 62 patients (69.66%) were diagnosed as asbestosis first, with a median time of 3.0 (IQR 1.0–10.5) years later diagnosed as malignant tumors.

**Table 1:** Demographics of enrolled patients.

Characteristics		All	With malignancies	Without malignancies	p-Value^*^
N (%)		544 (100%)	89 (16.4%)	455 (83.6%)	
Age, years		69.73 ± 8.76	69.43 ± 8.66	69.79 ± 8.79	0.72
Male gender, n (%)		240 (44.1%)	40 (44.9%)	200 (44.0%)	0.86
Exposure time, years		16.64 ± 12.00	17.42 ± 12.29	16.48 ± 11.95	0.50
Initial dust exposure age, years		18.32 ± 8.66	20.24 ± 9.29	17.94 ± 8.49	0.02
Latency, years		46.93 ± 11.17	44.84 ± 11.81	47.34 ± 11.01	0.05
BMI, kg/m^2^		26.05 ± 3.69	25.50 ± 3.22	26.12 ± 3.74	0.30
BMI^#^, kg/m^2^, n (%)	<18.5	7 (1.3%)	2 (2.2%)	5 (1.1%)	0.00
	18.5–24.9	160 (29.4%)	15 (16.9%)	145 (31.9%)	
	≥25.0	240 (44.1%)	27 (30.3%)	213 (46.8%)	
	Unknown	137 (25.2%)	45 (50.6%)	92 (20.2%)	
Smoking, n (%)		143 (26.3%)	29 (32.6%)	114 (25.1%)	0.14
Pack-years of smoking, n (%)	0	401 (73.7%)	60 (67.4%)	341 (74.9%)	0.03
	<10	37 (6.8%)	4 (4.5%)	33 (7.3%)	
	10–19.9	32 (5.9%)	7 (7.9%)	25 (5.5%)	
	≥20	74 (13.6%)	18 (20.2%)	56 (12.3%)	
Stages of asbestosis, n (%)	Stage I	434 (79.8%)	69 (77.5%)	365 (80.2%)	0.52
	Stage II	93 (17.1%)	19 (21.3%)	74 (16.3%)	
	Stage III	17 (3.1%)	1 (1.1%)	16 (3.5%)	

BMI, body mass index; N, number; SD, standard deviation.

Latency means the time of initial dust exposure year to diagnosis year. Exposure time means the time from the initial dust exposure year to the end of the dust exposure year. Data are presented as means ± SD or n (%).

*p-Values were calculated by t-test for continuous variables and Fisher’s exact test for categorical variables.

^#^The patients with BMI < 18.5 kg/m^2^ mean underweight, 18.5–24.9 kg/m^2^ mean normal range, and ≥25.0 kg/m^2^ mean overweight and obese.

Beijing used to be an industrial base of asbestos, and asbestos building materials were used in a large number of houses. The occupations of patients with asbestosis included 300 (55.1%) textile workers, 19 (3.5%) maintenance workers, 26 (4.8%) batchers, 12 (2.2%) brake pad producers, 20 (3.7%) building material producers, 19 (3.5%) boiler workers, and 148 (27.2%) other workers. The incidence of all malignancies and lung cancer had no significant difference between different work types. Malignancies occurred across all stages of asbestosis. We conducted a correlation analysis to investigate the relationship between the presence of malignancy and the severity of pneumoconiosis. Our findings indicated no significant association between asbestosis stage and malignancy occurrence.

No significant difference in lung function values was observed in the patients with or without malignancy (**Table S1**). The demographics of the enrolled patients according to the stages of chest image are shown in **Table S2**. Whether smoking or not, the patients with a higher stage of asbestosis had more airflow limitation, air trapping, and diffusion dysfunction. Forced vital capacity (%), forced expiratory volume in 1 s (%), TLC (%), and DLCO SB (%) were significantly reduced among the patients with different stages of asbestosis (**Table S3**).

### 3.2 Incidence of malignancies

**Figure 2:**
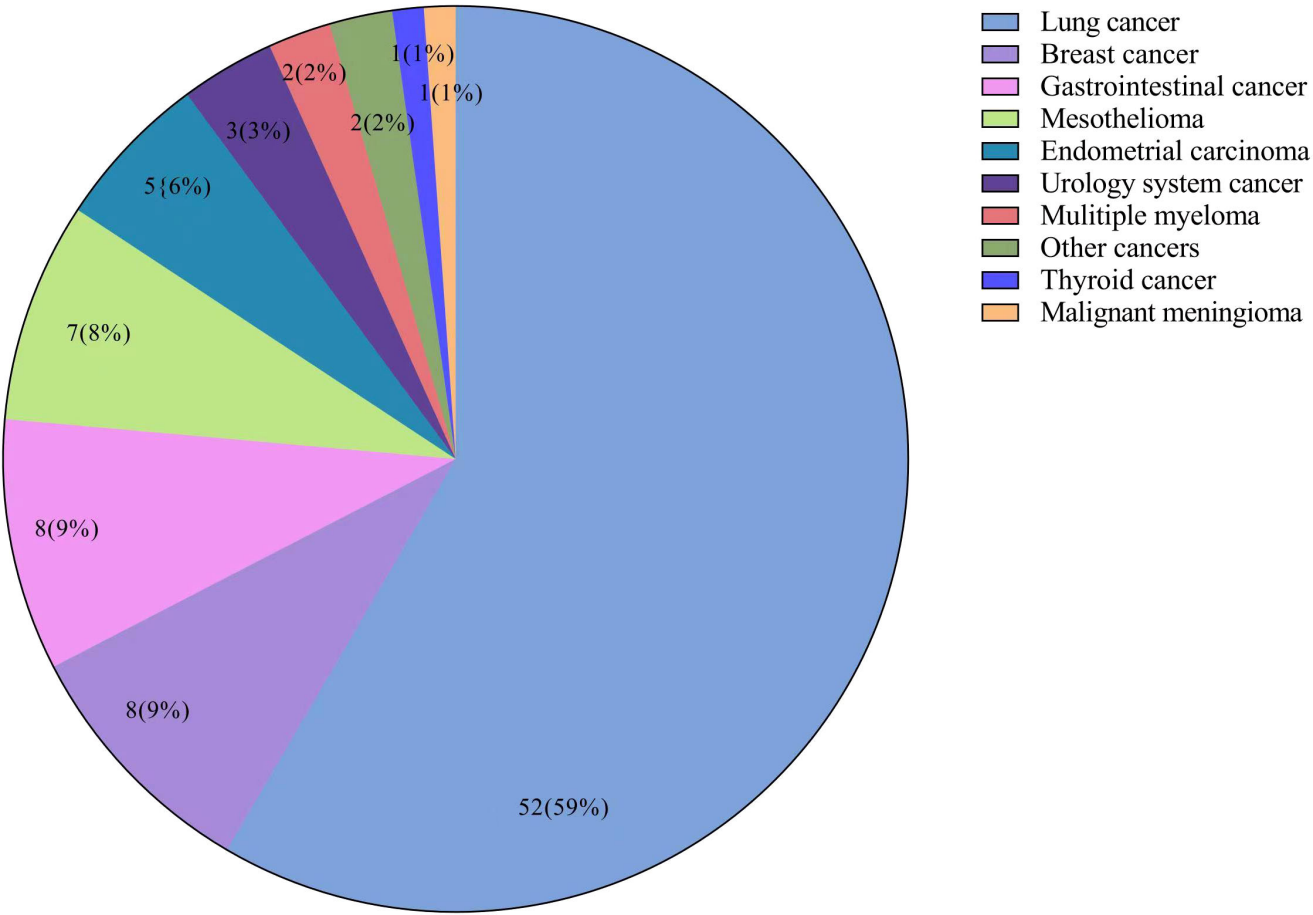
Distribution of malignancies in the patients with asbestosis (n = 89).

Of all 544 patients with asbestosis, a total of 89 (16.36%) patients had malignancies. The top four malignancies in asbestosis patients were lung cancer (52, 9.56%), breast cancer in female patients (8, 2.63%), gastrointestinal cancer (8, 1.47%), and mesothelioma (7, 1.29%). The composition of malignancies is shown in a pie chart (**Figure 2**). Among these patients with malignancies, four (4.49%) patients had two malignancies, which were endometrial carcinoma and lung cancer, endometrial carcinoma and breast cancer, kidney cancer and bladder cancer, and breast cancer and parafalcine meningiomas, respectively. In 52 patients with asbestosis and lung cancer, 33 (63.46%) had adenocarcinoma, 5 (9.62%) had squamous cell carcinoma, 4 (7.69%) had small cell carcinoma, and 10 (19.23%) had unknown pathological findings. The observed and expected incidence of malignancies and the corresponding SIRs in patients with asbestosis are shown in **Table 2**. A statistically significant increase in SIRs was noted for overall cancer (SIR 5.36, 95% CI 5.09–5.66), lung cancer (SIR 16.61, 95% CI 14.82–18.68), and mesothelioma (SIR 175, 95% CI 68.05–644.07). A statistically significant increase in breast cancer (SIR 5.23, 95% CI 4.39–6.26) and endometrial carcinoma (SIR 8.77, 95% CI 6.66–11.75) was found in female patients.

**Table 2:** Incidence of malignancies in the patients with asbestosis.

Malignancies	Observed (n)	Incidence (%)	Expected (n)	SIR* (%)	95% CI (%)
All malignancies^*^	89	16.36	16.59	5.36	5.09, 5.66
Lung cancer^*^(19)	52	9.56	3.13	16.61	14.82, 18.68
Mesothelioma^*^(20)	7	1.29	0.04	175.00	68.05, 644.07
Breast cancer^a*^(19)	8	2.63	1.53	5.23	4.39, 6.26
Endometrial carcinoma^a*^(19)	5	0.92	0.57	8.77	6.66, 11.75
Gastrointestinal cancer(19)					
Stomach cancer	1	0.18	1.63	0.61	0.47, 0.79
Colorectal cancer	5	0.92	1.57	3.19	3.01, 3.38
Pancreas cancer	2	0.37	0.38	5.33	4.77, 5.97
Genitourinary cancer					
Kidney cancer(21)	1	0.18	0.30	3.35	2.95, 3.83
Ureteral cancer(22)	1	0.18	0.06	15.74	14.53, 17.09
Prostate cancer^b^(23)	1	0.18	0.64	1.56	1.13, 2.17
Thyroid cancer(19)	1	0.18	1.05	0.95	0.72, 1.27
Malignant meningioma(19)	1	0.18	0.07	14.36	13.30, 15.54
Multiple myeloma(24)	2	0.37	0.49	4.07	3.69, 4.51
Others	2	/	/	/	/

SIR, standardized incidence ratio; CI, confidence interval.

The SIR is calculated based on the national cancer incidence rate in China (19). The other SIR is based on the latest published epidemiological data (20–24). Others: including malignant myxoma and ovarian cancer.

* Significant difference.

a Only female, n = 304.

b Only male, n = 240.

### 3.3 Risk factors of asbestosis-related malignancies

The median duration of chrysotile exposure was 12 years, ranging from 1 to 44 years. The median latent period was 50 years for patients with asbestosis and malignancies. The risk of all malignancies increased with initial exposure before 17 years old (HR 2.27, 95% CI 1.31–3.93, p = 0.01), longer asbestos exposure (HR 6.44, 95% CI 3.55–12.40, p < 0.01), and smoking (HR 1.66, 95% CI 1.00–2.67, p = 0.04) by Cox model fitted analysis (**Table 3**). The risk of lung cancer increased with smoking (HR 2.49, 95% CI 1.38–4.50, p = 0.01), initial exposure before 17 years old (HR 2.20, 95% CI 1.07–4.50, p = 0.03), and year of asbestos exposure (HR 10.83, 95% CI 4.64–25.27, p < 0.01), as shown in **Table 4**.

**Table 3:** Cox regression estimating hazard ratio and 95% confidence interval for malignancies.

	HR	95% CI	p-Value
Smoking status			
Smoking	1.66	1.00, 2.67	0.04
non-smokers	1.00	Ref.	
Age of initial dust exposure			
<17 years	2.27	1.31, 3.93	0.01
≥17 years	1.00	Ref.	
Latency			
>50 years	0.46	0.27, 0.77	0.01
≤50 years	1.00	Ref.	
Duration of asbestos exposure			
1–12 years	6.64	3.55, 12.40	0.00
12–44 years	1.00	Ref.	

HR, hazard ratio; CI, confidence interval.

Latency means the time of initial dust exposure year to diagnosis year. Duration of asbestos exposure means the time of initial dust exposure year to the end of dust exposure year.

**Table 4:** Cox regression estimating hazard ratio and 95% confidence interval for lung cancer.

	HR	95% CI	p-Value
Smoking status			
Smoking	2.49	1.38, 4.50	0.01
Non-smoking	1.00	Ref.	
Age of initial dust exposure			
<17 years	2.20	1.07, 4.50	0.03
≥17 years	1.00	Ref.	
Latency			
>50 years	0.47	0.24, 0.94	0.03
≤50 years	1.00	Ref.	
Duration of asbestos exposure			
1–12 years	10.83	4.64, 25.27	0.00
12–44 years	1.00	Ref.	

HR, hazard ratio; CI, confidence interval.

Latency means the time of initial dust exposure year to diagnosis year. Duration of asbestos exposure means the time of initial dust exposure year to the end of dust exposure year.

The risk of mesothelioma increased with the year of asbestos exposure (HR 7.38, 95% CI 1.24–43.97, p = 0.03). Asbestos exposure and tobacco smoking interact synergistically for the causation of various malignancies (HR 2.13, 95% CI 1.14–4.00, p < 0.01) or lung cancer (HR 2.61, 95% CI 1.22–5.60, p = 0.01), respectively, described by a multiplicative model. We found that longer asbestos exposure (HR 5.22, 95% CI 2.53–10.76, p < 0.01) increased the risk of all malignancies in women as well. There was no significant difference between gynecologic cancer, breast cancer, gastrointestinal cancer, and asbestosis exposure time. All models excluded smoke, sex, and age as confounders.

## 4 Discussion

ARDs are a serious public health threat and remain a major worldwide occupational burden. It is well known that asbestos exposure causes lung and pleural fibrosis (25). As a class I carcinogen, asbestos is closely related to the occurrence and development of malignancies (26). The cohort study is based on an active recruitment of long-term surveillance of patients with pneumoconiosis for the worker’s compensation. The data showed the incidence of malignancies in patients with asbestosis after being exposed to chrysotile in a median of 10 years’ longitudinal observation. A statistically significant increase in SIRs was observed for all malignancies. Among these, the SIRs of the asbestosis patients with malignancies were 16.61, 175.00, 5.23, and 8.77 in lung cancer, mesothelioma, breast cancer, and endometrial carcinoma, respectively. The duration of chrysotile exposure and history of smoking were independent risk factors for asbestosis combined with malignancies in this cohort. The carcinogenicity of chrysotile, especially for lung cancer while controlling for smoking, was analyzed. In addition, the data showed the detailed clinical characteristics of asbestos-induced healthy effects, such as asbestosis stages or lung function impairments, and the potential risk factors for the development of malignancies.

The association of exposure to chrysotile asbestos with lung cancer has been a pressing topic for a long time (27). The concentration of chrysotile remains stable and offers explanations for ARDs (28). A positive correlation between chrysotile and lung cancer is supported by increasing amounts of data, which is verified by our result as well (29, 30). The cohort studies of a Finnish population showed that the SIR of lung cancer was significantly increased in the asbestosis patients: the SIR for lung cancer was about twofold to 10-fold (31). Mortality rates for lung cancer and non-malignant respiratory diseases in asbestos workers were approximately four times higher than those of the general population (32, 33). Meanwhile, a strong exposure–response relationship between estimated exposure to asbestos and mortality from lung cancer and asbestosis has been found (34). Smoking and asbestosis have a joint effect on the increased incidence of lung cancer (35, 36). In comparison to the same period of a published cohort study, Chinese miners were exposed to much higher levels of asbestos overall than in Canada (32, 37). Data from the Qinghai mines indicated that total dust concentrations were 70 to 400 times higher than the national standard for asbestos dust (2 mg/m^3^) until 1995. In 2006, total stationary dust levels ranged from 12 to 197 mg/m^3^, averaging 91 mg/m^3^ (32). Meanwhile, Canadian studies revealed that workers with the highest exposure levels had an average exposure of 66.55 f/ml in 1968 when asbestos concentrations were at their peak, with this figure decreasing to 3.19 f/ml and the maximum exposure declining to 18.8 f/ml between 1976 and 1994 (37). The discrepancy between asbestos textile workers and asbestos miners was reported and evaluated in a number of studies, which suggested a generally higher risk of lung cancer in the asbestos textile industry than in the asbestos mining industry (38, 39). Due to economic reasons and ignorance of the hazards of asbestos, some of the individuals exposed to asbestos did not go through regular physical examinations, leading to a delayed diagnosis. Eventually, some of them developed symptoms of discomfort and were diagnosed with asbestos lung and malignant tumors at the same time at Beijing Chaoyang Hospital. The average latency period of these patients was 45.9 years, surpassing the typically accepted range of 10 to 40 years.

Mesothelioma, a “20th-century tumor”, is a rare malignancy with an incidence of four to five patients per 1,000,000 person/years, which develops in the pleura, pericardium, and peritoneum. Asbestos is the dominant cause of human malignant mesothelioma and is responsible for at least 85%–90% of pleural malignant mesotheliomas among men (40). The SIR of pleural mesothelioma was variable from 5.79 to 52.5 in workers exposed to asbestos (41, 42). Our data showed that SIR was much higher possibly due to the low incidence of mesothelioma in China. The incidence rate of mesothelioma ranges from 0.6 to 30 per million in Western countries (43), whereas the incidence of mesothelioma was only 1.5 per million in China from 2008 to 2012, which may be due to missing reports or underdiagnoses (44). Chrysotile may migrate from the lung to the pleura (45). An occupational history of brief or low-level exposure to asbestos was considered sufficient for mesothelioma to be considered occupationally related (20). Similar results were obtained here, which showed that longer exposure to chrysotile may confer a long-term risk of developing mesothelioma.

Evidence of a potential association between asbestos exposure and outcomes of gastrointestinal cancer has been observed. However, the data are inconsistent (46, 47). Drinking water contaminated with asbestos fibers from asbestos-containing water pipes or natural contamination has been noted (48). Studies among women exposed occupationally to various types of asbestos have reported increased risks for ovarian and cervical cancers, but not for breast cancer (49). The only study to suggest any association found a non-significant excess of breast cancer among female factory workers with severe exposure of 2 years’ duration (50). Asbestos fibers have been found in the ovaries of women whose household contacts worked with asbestos and among Norwegian paper and pulp workers (51). The mechanism of transportation of asbestos fibers to the ovary is not clearly understood. In the present study, there is no significant difference between gynecologic cancer, breast cancer, gastrointestinal cancer, and asbestosis exposure time. SIRs of gynecologic cancer, breast cancer, and gastrointestinal cancer were increased in asbestosis patients, which may partly be due to the screening effect. The relationships between asbestosis or asbestos exposure and various malignancies, e.g., gynecologic cancer, breast cancer, and gastrointestinal cancer, still need further investigation.

The screening effect during follow-up may indicate the early diagnosis of cancers in the cohort of asbestosis. A previous study showed that in 7.2 years of regular follow-up in Europe, the incidence of lung cancer was 0.49% (52). A 5-year, multicenter, population-based, prospective cohort study in China also showed that the incidence rate of lung cancer was 0.35% (53). In spite of a screening effect for lung cancer, the incident rate of lung cancer, as well as breast cancer and endometrial carcinoma, in patients of this cohort of asbestosis was higher than that in people without exposure according to the calculation of SIR for China (54, 55).

Despite these findings, the present study had several limitations. First, due to a lack of data on individual workplace environments or concentrations of asbestos fibers, this study may lack the ability to estimate the exposure–response relationship between cumulative exposure to asbestos and the prevalence of malignancies. Second, a selection bias may exist. A total of 544 patients with asbestosis were enrolled from a single medical center with a reputation for occupational medicine, and although the sample size is one of the largest in the country, the incidence data are still unknown for the entire nation. Third, the study was limited by the lack of a control group of dust-exposed workers without asbestosis, for comparison of the cancer incidence in patients with asbestosis and dust-exposed workers without asbestosis. Fourth, this study had a screening bias, which may lead to the early diagnosis or overdiagnosis of malignancies. The SIR of other unrelated cancers was increased, which may suggest that active cancer screening programs could have contributed to the increased detection of the whole range of malignancies. Finally, some of the malignancy sizes of the study population were too small to examine cancer types with less than one or two expected patients. The mortality of asbestosis remains to be elucidated in large samples and longitudinal observations.

## 5 Conclusions

The present study focused on the incidence rate of asbestosis combined with malignancies and other potential risk factors in a large Chinese cohort with median of 10 years’ follow-up. Asbestos exposure is not only a serious occupational health problem but also an environmental pollution that may continuously threaten the health of the population. The data demonstrated that the incidence of asbestosis combined with malignancies was greatly increased in lung cancer, mesothelioma, breast cancer, and endometrial carcinoma. The duration of asbestos exposure and history of smoking were related to the incidence of asbestosis with lung cancer. With comprehensive prevention and a complete prohibition of the production, processing, and use of asbestos, the malignancies resulting from asbestos exposure can be entirely avoided.

## Data availability statement

The original data presented in the study are included in the article/**Supplementary Material**. Further inquiries can be directed to the corresponding author.

## Ethics statement

The studies involving human participants were reviewed and approved by the Institutional Review Board of Beijing Chaoyang Hospital. The patients/participants provided their written informed consent to participate in this study.

## Author contributions

Conceptualization: QY. Data curation: JW, RM, and XH. Formal analysis: JW, RM, and XH. Funding acquisition: QY. Investigation: JW, RM, NW, and XD. Supervision: QY. Writing—original draft preparation: JW. Writing—review and editing: QY. All authors contributed to data interpretation. All authors contributed to the article and approved the submitted version.
