# Supplementary material for: The incidence of malignancies in asbestosis with chrysotile exposure: a large Chinese prospective cohort study
Source: Front Oncol. 2023 Jul 6;13:1172496. doi: 10.3389/fonc.2023.1172496 (PMC10359706; doi:10.3389/fonc.2023.1172496)
Supplement: Supplementary file 1 [file DataSheet_1.pdf]

Supplementary Material

The incidence of malignancies in asbestosis with chrysotile exposure: a large Chinese prospective cohort study

1 Supplementary Data

1.1 Methods

1.1.1 Data collection

Smoking status was self-reported and classified as smoker or never smoker. Smoking intensity was analysed as both a categorical (non-smokers, <10 pack-years, 10 to 19 pack-years, and ⩾20 pack-years) and continuous variables. Both categorical (<18.5 kg/m^2, 18.5 to 24.9 kg/m^2, and ⩾25.0 kg/m^2) and continuous variables to analyze body mass index were also used for analysis.

1.1.2 Chest radiograph classification

Two occupational medicine experts independently evaluated the images in accordance with the International Labour Organization classification system (1). Pneumoconiosis is classified into three stages according to chest X-ray radiographs (2). Briefly, the lung fields were divided into six zones on a posterior chest radiograph. When the highest density of small opacities was ⩾1/0, the distribution affected two or more zones and pleural plaques were apparent, and the patients were diagnosed with stage I disease. When the highest density of small opacities was ⩾2/1 and the distribution affected more than four zones, or the highest density of small opacities was ⩾3/2 and the distribution affected four or more zones, the patients were diagnosed as stage II. When the highest density of small opacities was ⩾3/2 and the distribution affected four or more zones with aggregation of small opacities, the patients were diagnosed as stage III. The interobserver correlation was good, with a value of 0.82.

1.1.3 Malignancies diagnoses

The diagnosis of lung cancer and mesothelioma related to asbestos was based on the Helsinki criteria for occupational malignancies (3). The diagnosis of breast cancer (4), endometrial cancer (5), colorectal cancer (6), gastric cancer (7), pancreatic cancer (8), prostate cancer (9), ureteral cancer (10), renal cell carcinoma (11), thyroid cancer (12), multiple myeloma (13), malignant meningioma (14) and other malignancies were consistent with the latest guidelines.

1.1.4 **Sample size calculation**

Based on a previous study, this study assumed that the prevalence of malignancies in asbestosis was 30.59%. Using the formula, the sample size was 533 (15). If fixed the two-sided 95% confidence interval width was 0.01, the sample size was 344. We calculated sample sizes with PASS software (NCSS, Kaysville, UT, USA).

1.1.5 Hospital selection

Beijing Chaoyang Hospital has a regional center for occupational medicine and worker's compensation. Patients with pneumoconiosis who have ever worked or lived in the city or been transferred from other regions will come to the hospital as outpatients or inpatients. The majority of the patients with asbestosis were from a district on the east of the city with the asbestos products factories ever opened from 1950s to 1970s located 20 kilometers away from the hospital.

This study is based on an active recruitment of a long-term surveillance of the patients with pneumoconiosis for the worker’s compensation. All the reported pneumoconiosis patients with compensation have participated the surveillance plan. Prior to the commencement of the research, participants who had already been diagnosed were contacted by telephone, to be followed up at the clinic every 12 months. A clinical pathway was used for the diagnosis and evaluation of the disease and for the worker’s compensation. All recruited patients at Beijing Chaoyang Hospital have complete inpatient and/or outpatient records, as well as various examinations. In cases where complete medical records were unavailable at our hospital, we conducted interviews with patients to obtain whole copies of the medical records from other hospitals. These medical records served as the basis for occupational disease identification and work-related injury compensation. Once diagnosed with asbestosis, the patients will undergo regular follow-up and condition monitoring in accordance with regulations, in order to facilitate clinical care and receive further compensation for work-related injuries. To the best of our knowledge, the Beijing Chaoyang Hospital had the largest cohort of asbestosis in China.

1.1.6 The calculation of SIR

After careful consideration, the national cancer incidence rate was selected to calculate the Standardized Incidence Ratio (SIR). This decision was based on the fact that Beijing does not have comprehensive and systematic statistics on various types of tumors, with some cancer types being incompletely reported and data quality being suboptimal. Additionally, Beijing is a megacity with a large floating population from other provinces and cities, amounting to over 11 million people daily. This creates substantial difficulties with data statistics. Moreover, the data from Beijing generally only accounts for urban areas, while many asbestosis patients live in rural areas, resulting in a significant divergence between the incidence rate and data from Beijing.

1.2 Supplementary References

1. International Labour Office. International Classification of Radiographs of Pneumoconiosis, revised. Occupational Safety and Health Series (2011) 22:Rev 2011.

2. National occupational health standard. Diagnosis of occupational pneumoconiosis (GBZ 70-2015). (2015). http://www.nhc.gov.cn/wjw/pyl/wsbz.shtml. (Accessed January 31 2023).

3. Wolff H, Vehmas T, Oksa P, Rantanen J, Vainio H. Asbestos, asbestosis, and cancer, the Helsinki criteria for diagnosis and attribution 2014: recommendations. Scand J Work Environ Health (2015) 41 (1): 5–15.

4. Gradishar WJ, Moran MS, Abraham J, Aft R, Agnese D, Allison KH, et al. NCCN Guidelines® Insights: Breast Cancer, Version 4.2021. J Natl Compr Canc Netw (2021) 19 (5): 484–493.

5. Abu-Rustum NR, Yashar CM, Bradley K, Campos SM, Chino J, Chon HS, et al. NCCN Guidelines® Insights: Uterine Neoplasms, Version 3.2021. J Natl Compr Canc Netw (2021) 19 (8): 888–895.

6. Benson AB, Venook AP, Al-Hawary MM, Arain MA, Chen YJ, Ciombor KK, et al. Colon Cancer, Version 2.2021, NCCN Clinical Practice Guidelines in Oncology. J Natl Compr Canc Netw (2021) 19 (3): 329–359.

7. Ajani JA, D'Amico TA, Almhanna K, Bentrem DJ, Chao J, Das P, et al. Gastric Cancer, Version 3.2016, NCCN Clinical Practice Guidelines in Oncology. J Natl Compr Canc Netw (2016) 14 (10): 1286–1312.

8. Tempero MA, Malafa MP, Al-Hawary M, Behrman SW, Benson AB, Cardin DB, et al. Pancreatic Adenocarcinoma, Version 2.2021, NCCN Clinical Practice Guidelines in Oncology. J Natl Compr Canc Netw (2021) 19 (4): 439–457.

9. Schaeffer E, Srinivas S, Antonarakis ES, Armstrong AJ, Bekelman JE, Cheng H, et al. NCCN Guidelines Insights: Prostate Cancer, Version 1.2021. J Natl Compr Canc Netw (2021) 19 (2): 134–143.

10. Flaig TW, Spiess PE, Agarwal N, Bangs R, Boorjian SA, Buyyounouski MK, et al. Bladder Cancer, Version 3.2020, NCCN Clinical Practice Guidelines in Oncology. J Natl Compr Canc Netw (2020) 18 (3): 329–354.

11. Motzer RJ, Jonasch E, Boyle S, Carlo MI, Manley B, Agarwal N, et al. NCCN Guidelines Insights: Kidney Cancer, Version 1.2021. J Natl Compr Canc Netw (2020) 18 (9): 1160–1170.

12. Haddad RI, Nasr C, Bischoff L, Busaidy NL, Byrd D, Callender, G, et al. NCCN Guidelines Insights: Thyroid Carcinoma, Version 2.2018. J Natl Compr Canc Netw (2018) 16 (12) : 1429–1440.

13. Kumar SK, Callander NS, Adekola K, Anderson L, Baljevic M, Campagnaro E, et al. Multiple Myeloma, Version 3.2021, NCCN Clinical Practice Guidelines in Oncology. J Natl Compr Canc Netw (2020) 18 (12): 1685–1717.

14. Goldbrunner R, Minniti G, Preusser M, Jenkinson MD, Sallabanda K, Houdart E, et al. EANO guidelines for the diagnosis and treatment of meningiomas. Lancet Oncol (2016) 17 (9): e383–e391.

15. Oksa P, Klockars M, Karjalainen A, Huuskonen MS, Vattulainen K, Pukkala E, et al. Progression of asbestosis predicts lung cancer. Chest (1998) 113(6), 1517-21.

2 Supplementary Figures and Tables

Table S1 Lung function values of the patients with and without malignancies in smoking and non-smoking asbestosis

Parameters	All	With malignancies	Without malignancies	*P*-value
Non-smokers				
FVC, L	1.96±0.66	1.79±0.60	1.99±0.67	0.13
FVC, %pred	77.70±23.76	76.06±24.20	77.89±23.75	0.69
FEV_1, L	1.52±0.55	1.40±0.51	1.54±0.56	0.22
FEV_1, %pred	74.24±24.37	72.31±24.10	74.45±24.43	0.67
FEV_1/FVC, %	76.03±14.30	76.43±13.41	75.98±14.41	0.88
TLC, L	4.10±4.12	4.42±4.30	4.07±4.11	0.68
TLC, %pred	77.41±23.68	78.97±24.02	77.24±23.69	0.72
DLCO, %pred	62.25±25.49	64.97±22.90	61.94±25.80	0.55
PaO2, mmHg (room air, at rest)	83.83±20.07	85.56±23.88	83.53±19.37	0.52
Smokers				
FVC, L	2.58±0.83	2.53±0.72	2.59±0.85	0.79
FVC, %pred	73.74±23.38	71.21±17.00	74.26±24.53	0.59
FEV_1, L	1.94±0.69	1.65±0.47	1.97±0.71	0.11

FEV_1, %pred	71.33±25.10	64.25±19.28	72.23±25.69	0.28
FEV_1/FVC, %	75.31±12.97	71.72±12.89	75.80±12.96	0.27
TLC, L	4.72±3.71	4.18±0.75	4.79±3.93	0.58
TLC, %pred	72.53±22.30	66.99±17.65	73.24±22.80	0.34
DLCO, %pred	65.49±26.39	64.22±23.05	65.75±27.12	0.81
PaO2, mmHg (room air, at rest)	82.20±15.60	80.46±15.01	82.72±15.82	0.53

Abbreviations: DLCO, diffusing capacity of the lung for carbon monoxide; FEV_1, forced expiratory volume in the first second; FVC, forced vital capacity; PaO2, the arterial partial pressure of oxygen; TLC, total lung capacity;

Data were present as mean±SD or n(%).

Table S2 Demographics of the enrolled patients according to stages of asbestosis

Parameters		All	Stage I	Stage II	Stage III	*P*-value
n (%)		544(100%)	434(79.8%)	93(17.1%)	17(3.1%)	
Age, yrs		69.73±8.76	69.90±8.78	69.35±8.68	67.53±8.95	0.77
Male gender, n (%)		240(44.1%)	187(43.1%)	45(48.4%)	8(47.1%)	0.63
Exposure time, yrs		16.64±12.00	17.01±11.91	16.00±12.54	10.53±9.82	0.29
Initial dust exposure age, yrs		18.32±8.66	18.72±8.87	16.69±7.16	16.82±9.98	0.57
Latency*, yrs		46.93±11.18	46.26±11.44	49.61±9.40	49.41±11.11	0.17
BMI, kg/m^2		26.05±3.69	26.19±3.64	25.98±3.82	23.71±3.41	0.03
BMI#, kg/m^2 n (%)	<18.5	7(1.3%)	3(0.7%)	4(4.3%)	0	0.00
	18.5-24.9	160(29.4%)	123(28.3%)	27(29.0%)	10(58.8%)	
	≥25.0	240(44.1%)	185(42.6%)	49(52.7%)	6(35.3%)	
	unknown	137(25.2%)	123(28.3%)	13(14.0%)	1(5.9%)	
Smoking, n (%)		143 (26.3%)	107(24.7%)	31(33.3%)	5(29.4%)	0.21
Smoking pys, n (%)	0	401(73.7%)	327(75.3%)	62(66.7%)	12(70.6%)	0.43
	<10	37(6.8%)	27(6.2%)	8(8.6%)	2(11.8%)	
	10-19	32(5.9%)	26(6.0%)	5(5.4%)	1(5.9%)	
	⩾20	74(13.6%)	54(12.4%)	18(19.4%)	2(11.8%)	

Malignancies, n (%)	89(16.4%)	69(15.9%)	19(20.4%)	1(5.9%)	0.28

Abbreviations: BMI, body mass index; N, Number; SD, standard deviation.

Data are present as means±SD or n(%).

P-value were calculated by analysis of variance for continuous variables and *Fisher's* exact test for categorical variable.

*Latency means the time of initial dust exposure year to diagnosis year; Exposure time means the time of initial dust exposure year to the end of dust exposure year.

#The patients with BMI <18.5 kg/m^2 mean underweight, 18.5-24.9 kg/m^2 mean normal range, and ≥25.0 kg/m^2 mean overweight and obese

Table S3 Lung function parameters of the enrolled patients according to stages of asbestosis

Parameters	All	Stage I	Stage II	Stage III	*P*-value
Non-smokers					
FVC, L	1.96±0.66	2.07±0.64	1.60±0.57	1.65±0.74	0.00
FVC, %pred	77.70±23.76	81.15±23.93	67.78±19.16	60.46±20.53	0.00
FEV_1, L	1.52±0.55	1.62±0.54	1.21±0.47	1.27±0.64	0.00
FEV_1, %pred	74.24±24.37	77.97±24.37	63.16±19.39	58.21±24.50	0.00
FEV_1/FVC, %	76.03±14.30	76.02±15.15	76.05±10.17	76.12±15.62	0.53
TLC, L	4.10±4.12	4.15±4.10	4.05±4.61	3.43±1.08	0.80
TLC, %pred	77.41±4.12	79.64±22.83	70.48±26.63	69.05±17.39	0.01
DLCO, %pred	62.26±25.49	67.26±24.03	46.75±23.68	40.64±20.99	0.00
PaO2, mmHg (room air, at rest)	83.83±20.07	85.72±19.57	77.65±19.42	74.59±26.09	0.01
Smokers					
FVC, L	2.58±0.83	2.69±0.88	2.32±0.51	1.69±0.32	0.01
FVC, %pred	73.74±23.38	77.03±23.75	63.72±17.90	59.10±26.95	0.02
FEV_1, L	1.94±0.69	2.03±0.73	1.71±0.46	1.33±0.26	0.01

FEV_1, %pred	71.33±25.10	74.59±24.85	61.41±23.31	59.88±28.58	0.03
FEV_1/FVC, %	75.31±12.97	75.90±10.28	72.59±20.71	78.71±3.56	0.87
TLC, L	4.72±3.71	4.99±4.18	4.00±1.22	3.13±0.41	0.39
TLC, %pred	72.53±23.30	75.83±20.84	66.91±21.35	34.53±21.64	0.00
DLCO, %pred	65.49±26.39	70.92±25.48	48.99±22.15	40.93±16.20	0.01
PaO2, mmHg (room air, at rest)	82.20±15.60	84.26±14.99	77.16±16.85	68.50±7.55	0.03

Abbreviations: PaO2, the arterial partial pressure of oxygen; FVC, forced vital capacity; FEV_1, forced expiratory volume in the first second; TLC, total lung capacity; DLCO, diffusing capacity of the lung for carbon monoxide.

Data are present as means±SD or n(%).
